# Automating Radiographic Measurements of the Hip in Children with Cerebral Palsy

**DOI:** 10.1302/0301-620X.107B1.BJJ-2024-0894

**Published:** 2025-01-01

**Authors:** Peter Thompson, Mohammed Khattak, P. Josiah Joseph, Daniel C. Perry, Medical Annotation Collaborative, Dileep Karthikappallil, Hesham Zaman, Grace Airey, Saad Maqsood, Tom Hughes, Shuja Ahmad, James McEvoy, Graeme Wilson, Ha Phuong Do Le, Fatima Tariq, Sohan Shah, Dhawal Patel, Ross McAllister, Anil Singh Dhadwal, Joseph Fennelly, William Lloyd, Amir Varasteh, Kieran Almond, Henry Crouch-Smith, Timothy F. Cootes, Claudia Lindner

**Affiliations:** 1 Division of Informatics, Imaging & Data Sciences, The University of Manchester, United Kingdom; 2 University of Liverpool, United Kingdom; 3 Alder Hey Children’s Hospital, Liverpool, United Kingdom

## Abstract

**Aims:**

The aims of this study were (i) to develop an automatic system capable of calculating four radiographic measurements used in the diagnosis and monitoring of cerebral palsy (CP) related hip disease, and (ii) to demonstrate that these measurements are sufficiently accurate to be used in clinical practice.

**Methods:**

We developed a machine-learning system to automatically measure Reimer’s migration percentage (RMP), acetabular index (AcI), head shaft angle (HSA) and neck shaft angle (NSA). The system automatically locates points around the femoral head and acetabulum on pelvic radiographs, and uses these to calculate measurements. The system was evaluated on 1650 pelvic radiographs of children with CP (682/968 females/males, mean age: 8.3±4.5 SD years). Each radiograph was manually measured by five clinical experts. Agreement between the manual clinical measurements and the automatic system was assessed by mean absolute deviation (MAD) from the mean manual measurement, type-1 and type-2 intra-class correlation coefficients (ICC1, ICC2), and a linear mixed-effects model (LMM) for assessing bias.

**Results:**

The MAD scores were 5.7% (SD: 8.5%) for RMP, 4.3° (SD: 5.4°) for AcI, 5.0° (SD: 5.2°) for NSA, and 5.7° (SD: 6.1°) for HSA. Overall ICC scores quantifying the agreement between the mean manual measurement and the automatic results were 0.91 for RMP, 0.66 for AcI, 0.85 for NSA, and 0.73 for HSA. The LMM showed no statistically significant bias.

**Conclusion:**

The results showed excellent agreement between the manual and automatic measurements for RMP, good agreement for NSA, and moderate agreement for HSA and AcI. The performance of the system is sufficient for application in clinical practice to support the assessment of hip migration based on RMP. The system has the potential to save clinician’s time, and to improve patient care by enabling more comprehensive, consistent, and reliable monitoring of hip migration in children with CP.

## 1 Introduction

One third of children with Cerebral palsy (CP) have hip problems, and detecting early signs of hip disease is key to the effectiveness of treatment^1^. The amount of hip migration and rate of progression of hip migration are key risk factors for hip dislocation^2^.

Hip surveillance programmes monitor children with CP for signs of hip disease and have been shown to effectively reduce the frequency of hip dislocation, with improved clinical outcomes^3,4,5^. However, assessing radiographic signs of hip disease in children affected by CP is an additional task for busy clinicians. Consequently, meticulously recording measurements and uploading the results to surveillance systems is frequently not performed, or is delegated to less experienced staff. This minimises the ability to reliably track hip migration and introduces inequalities depending on the local engagement of clinicians with the surveillance programme.

An automatic system to record radiographic measurements has the potential to improve the consistency of care for children with CP-related hip disease, to enable more timely interventions and to save clinicians’ time.

The aim of this study was to develop and evaluate an automatic software system to calculate Reimer’s migration percentage (RMP), acetabular index (AcI), femoral neck shaft angle (NSA) and femoral head shaft angle (HSA), four radiographic measurements used in the diagnosis and monitoring of CP-related hip disease. This work is based on a preliminary study^6^ where we demonstrated a proof-of-concept for automatically measuring AcI and RMP in a non-CP paediatric population.

## 2 Methods

### 2.1 Data

A database of 3435 anonymised paediatric pelvic radiographs of children with CP was retrospectively collected from Alder Hey Children’s Hospital. All images had been previously acquired as part of clinical care. Research ethics approval was not required as the data was completely anonymised. Data was released with the permission of the Alder Hey Hospital Caldecott guardian.

**Figure 1:**
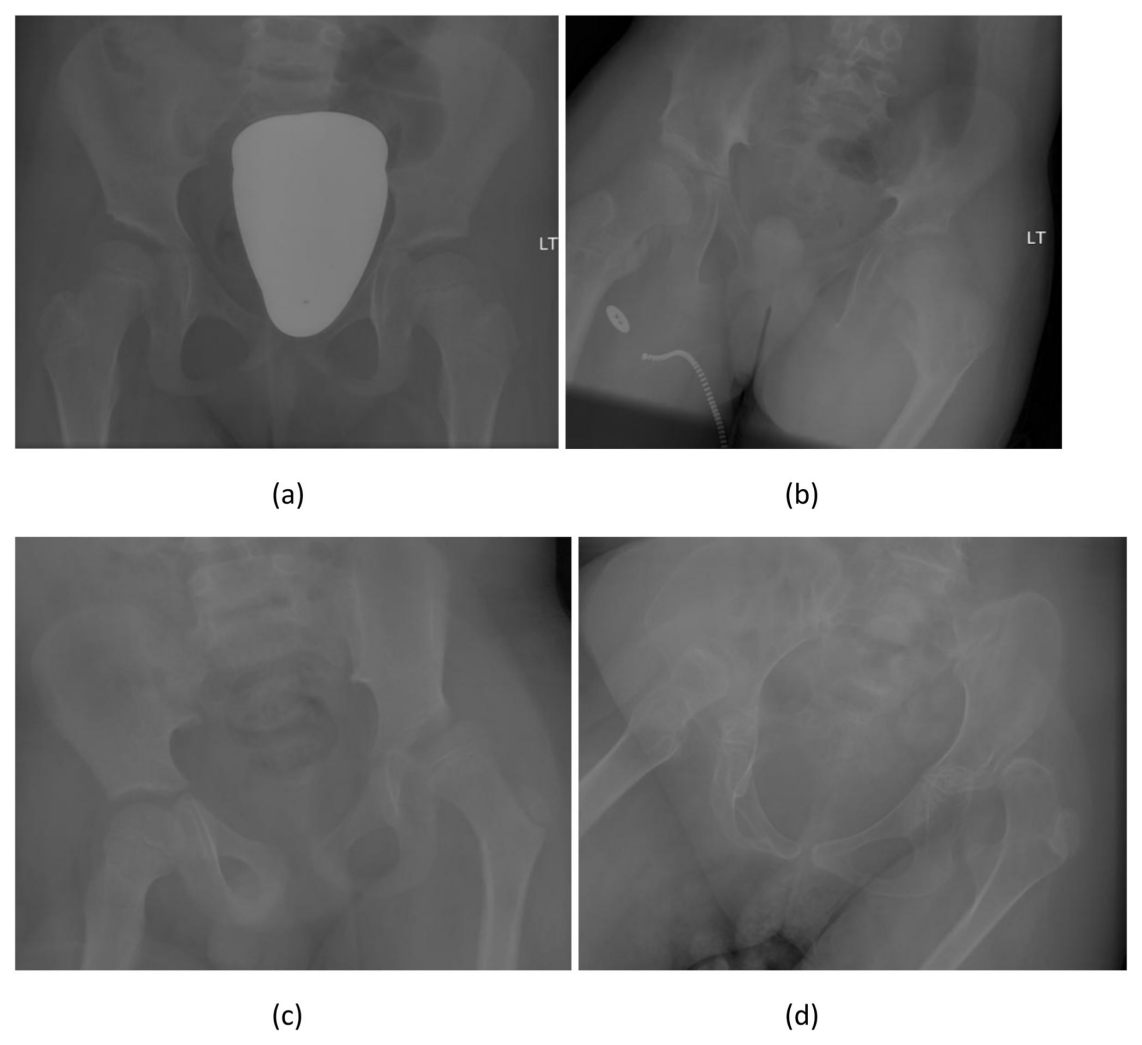
Example left hips across a range of severities: (a) no migration, (b) possible subluxation, (c) definite subluxation, (d) dislocation

Images were excluded if one or both hips were not visible in the image due to framing or artefacts (n=153), the radiograph not being a pelvic AP image (n=35), very poor positioning (n=95), signs of previous surgical intervention (n=784), image duplication (n=7), or the hip was dislocated and the femur was overlapping the pelvis (n=31). The remaining 2330 images were divided into four categories based on visual signs of hip migration: no signs of migration, possible subluxation, clear subluxation, and dislocation. This was done to randomly select 50 images that represent the study population, sampling 11-13 hips for each of the four categories. An example hip from each of these categories is shown in Figure 1. These 50 images were the universal dataset, which was included in all measurement subsets (see below). A further 1600 images were randomly selected. The number of left hips in each severity category was 1182 (no migration), 128 (possible subluxation), 119 (clear subluxation), 171 (dislocation), and for the right hips it was 1168 (no migration), 122 (possible subluxation), 144 (clear subluxation), 166 (dislocation). We were not able to include the remaining images due to capacity constraints for getting all images manually measured (see below). The total CP dataset comprised 1650 images, 682 female and 968 male, with a mean age of 8.3 years (SD: 4.5).

The CP dataset was split into four subsets (Sets 1-4) to be manually measured by clinical experts (Medical Annotation Collaborative; specialist orthopaedic trainee doctors that had undergone formal training to ensure a standardised level of expertise). Each subset contained the 50 images from the ‘universal set’ and 400 from the ‘representative sample’. Each subset was analysed by five experts, with the aid of an in-house software tool that enabled them to place the lines defining the measurements, similar to those shown in Figure 3. RMP, AcI and NSA were measured for all four sets, while HSA was added later and thus was measured for one set only (Set 4).

To increase the training set for developing the automatic system, we also included the 450 paediatric pelvic radiographs from our previous study^6^. These were of 150 children with developmental dysplasia of the hip (DDH), 150 children with Perthes disease, and 150 healthy subjects.

### 2.2 Automatic point placements

**Figure 2:**
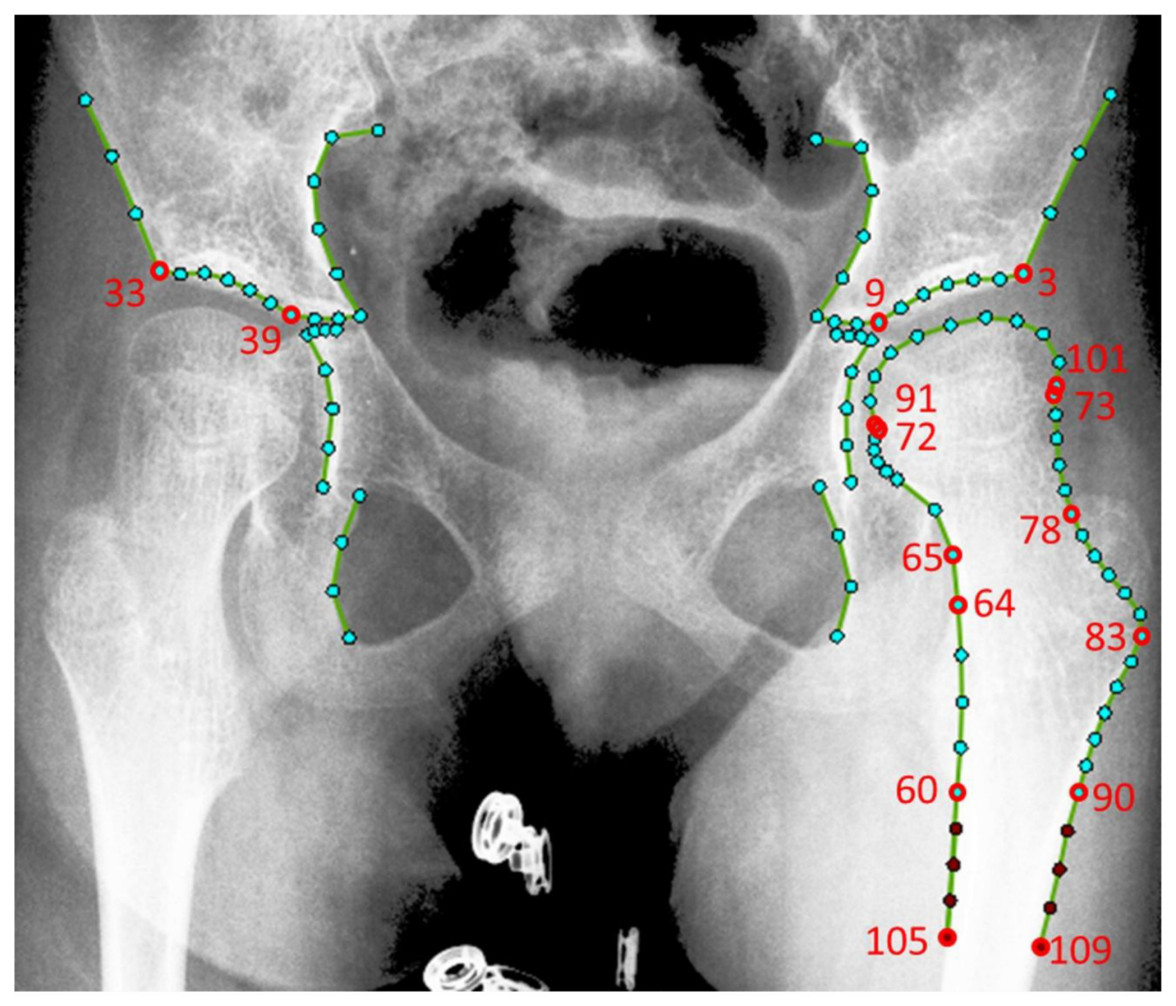
An example of the 110-point model for measuring the anatomical left hip. The additional points included in the extended model are highlighted in red. Points 9 and 39 define Hilgenreiner’s line. A line of best fit drawn through points 3 to 9 defines the acetabular roof. Point 3 defines the end of the acetabular roof, with Perkin’s line being that which is perpendicular to Hilgenreiner’s line and passes through this point. Points 91 to 101 define the femoral head, with the bounds being taken to be the points of maximum separation parallel to Hilgenreiner’s line. The midpoints of points 64 and 83 and points 60 and 90 (or 105 and 109 in the extended case) define the femoral shaft axis. The midpoints of points 72 and 73 and points 65 and 78 define the femoral neck shaft axis. The line from point 72 to 73 was taken to approximate the epiphyseal plate axis.

The automatic system is based on a technology called Random Forest Regression Voting in the Constrained Local Model framework (RFRV-CLM)^7^. This is a supervised machine-learning approach that enables point positions to be located in images with no human input. An example of the point positions used in this study is shown in Figure 2. These include 60 points to mark distinctive features of the pelvis, including the triradiate cartilage and acetabular roof, and 50 points to outline the proximal femur. For training the automatic system, we manually annotated all 1650 images with all point positions, and also had access to the manual annotations for the 450 images from our prior work on pelvic radiographs affected by DDH^6^.

A fully automatic system based on RFRV-CLM works in two stages: a global stage that searches the whole image for candidate regions of interest, and a local stage that iteratively searches for the best possible configuration of points, in terms of both the relevance of the local image features and the distribution of point locations^8^.

We trained the system using all 2100 images. Full details on how to train such system are given elsewhere^7,8^, here we describe our experimental set-up. We used a single global stage that searches for the region of the pelvis. Our local stage consisted of three phases. The first coarsely fits all points in the model, and the subsequent two independently fit the pelvis (points 0-59) and femur (points 60+). This approach was chosen to ensure the femur is positioned in roughly the correct position, relative to the pelvis, without being overly restricted, particularly in cases of severe subluxation and dislocation.

We used a three-fold cross-validation approach to obtain the automatic point positions for our CP dataset. Cross-validation experiments are systematic leave-some-out experiments in which a dataset is divided into a number of subsets (folds), and then the system is trained on all but one fold and tested on the remaining fold. The process is repeated until all folds have been tested. This approach allowed us to use all our data for both training the systems and obtaining automatic point positions, without using the same data for training and testing.

### 2.3 Measurements

**Figure 3:**
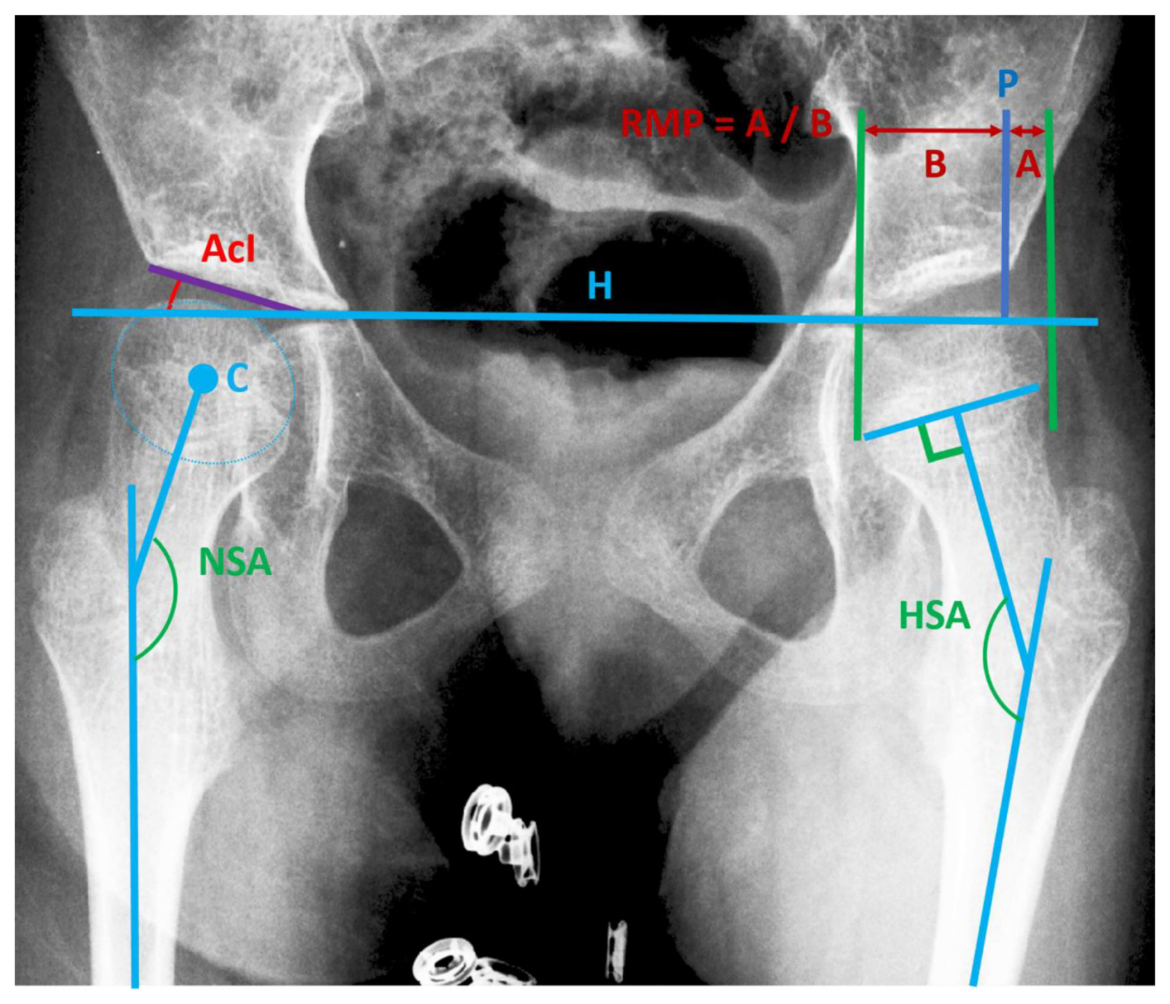
Diagram showing how the four measurements, RMP, AcI, NSA and HSA, are defined for an example radiograph. Lines H and P are Hilgenreiner’s and Perkin’s line, respectively.

A diagram demonstrating the four measurements, RMP, AcI, NSA and HSA, is shown in Figure 3. Both RMP and AcI were measured using a reference line drawn through the triradiate cartilage on each side of the pelvis, also referred to as Hilgenreiner’s line. AcI was the angle between Hilgenreiner’s line and the slope of the acetabular roof. To calculate RMP, three lines were drawn perpendicular to Hilgenreiner’s line, one from the edge of the acetabular roof, referred to as Perkin’s line, and two outlining the edge of the femoral head. RMP was the proportion of femoral head that extends beyond Perkin’s line. NSA was the angle between the femoral shaft axis and the neck shaft axis, with the neck shaft axis calculated as the line that passes through the centre of the femoral head and the femoral neck shaft at its narrowest point. HSA was the angle between the femoral shaft axis and the line drawn perpendicular to the edges of the epiphyseal plate.

For the automatic system, the point positions obtained from the RFRV-CLM were used to automatically calculate the measurements using the software tool BoneFinder®. Figure 2 provides details on what role specific point positions had in the calculations.

### 2.4 Evaluation

We developed and evaluated three systems:

- **Non-extended:** This system included a RFRV-CLM to automatically position 102 points (excluding the femoral shaft points highlighted in red in Figure 2).
- **Extended:** This system included a RFRFV-CLM to automatically position all 110 points as per Figure 2.
- **Extended-fast:** This system included a RFRV-CLM to automatically position the same points as the extended model but it was run-time optimised to speed up the search to obtain the automatic point positions. This was achieved by the system stopping the search when it assessed it had found a good enough result.

For each system, we applied three-fold cross-validation experiments as described above to obtain three sets of automatic point positions for each image, one for each system. For each set of automatic point positions, the measurements were then automatically generated using BoneFinder®.

We used several methods to assess the agreement between the manual measurements and the measurement results of the automatic systems for the CP dataset:

- The mean absolute deviation (MAD), which was the average absolute difference between the mean manual measurement and the automatic measurement for each hip.
- The intra-class correlation coefficient between the mean manual and automatic measurements. Both the type-1 (ICC1) and type-2 (ICC2) coefficients were used. ICC2 was used to assess the agreement within a set (i.e. when the measurements were made by the same five raters) and on the 50 images that were shared between the sets. ICC1 was used to assess the overall agreement across all four sets. ICC scores are usually interpreted as poor below 0.5, moderate below 0.75, good below 0.9 and excellent from 0.9^9^.
- A linear mixed-effects model (LMM) was used to verify that the automatic measurements had no statistically significant bias relative to the distribution of manual measurements.
- The diagnostic consistency between the mean manual and automatic RMP measurements was calculated for diagnostic thresholds of 30%, 40% and 50%. These thresholds were motivated by current management around monitoring hip migration in children with CP, which often use an RMP>30% to initiate a referral to the orthopaedics department and an RMP>40% to consider surgical interventions^10^. An RMP>50% has recently been identified as the ‘point of no return’, indicating that an affected hip would not return to normal without surgical intervention^11,12^.

## 3 Results

Best performance was achieved for the extended system, followed closely by the extended-fast system (see MAD results, Table 1). Extending the femoral shaft points led to a clear improvement in measuring NSA and HSA, compared to the non-extended system.

Table 2 shows the ICC2 scores quantifying the agreement within the clinical experts as well as between the mean clinical expert manual measurements and the automatic system (evaluated for each of the three systems). ICC2 values were similar across the four subsets as well as the universal shared dataset, indicating that subset composition and annotator performance was not biased. The ICC1 scores of an overall assessment of the three systems across all images is given in Table 3. The results demonstrate that we achieve excellent agreement for RMP, good agreement for NSA, and moderate agreement for HSA and AcI. A similar pattern can be seen regarding agreement amongst the clinical experts. The lowest ICC scores were achieved for AcI with significantly less agreement amongst clinical experts compared to the agreement between the experts with the extended system.

**Figure 4:**
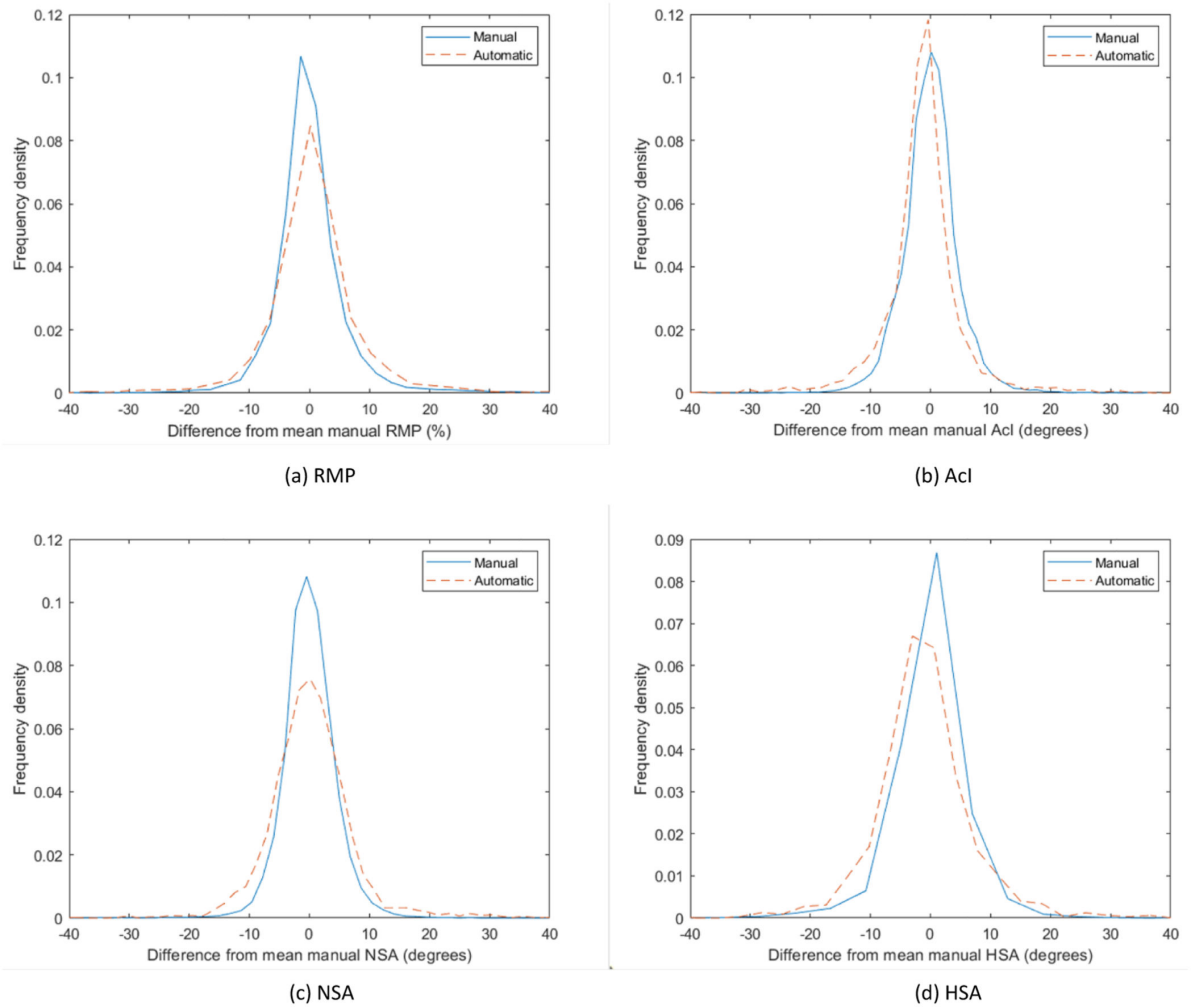
Histograms showing the distribution of differences from the mean manual measurement for the clinicians and the automatic measurements (extended system) across Sets 1-4 (1650 images, left and right hips) for RMP, AcI, NSA and across Set 4 (450 images, left and right hips) for HSA. The area under the curves was normalised to be one in each case. For ease of visualisation, the centres of the bins are visualised as connected curves.

The results obtained from the LMMs (Table 4) show that there is no significant bias between the manual and automatic measurements for the extended systems, suggesting that the clinical expert measurements and the automatic measurements are in agreement. This is also confirmed by the histograms in Figure 4 which show that the distribution of differences from the mean manual measurement for the automatic measurements is similar to that of the individual clinical experts.

The diagnostic consistency results for informing treatment decisions based on RMP are shown in Table 5. The results demonstrate high consistency with sensitivities above 0.9 for all thresholds, and specificities above 0.85/0.9/0.9 for the 30%/40%/50% thresholds for all three systems. The extended system was able to identify hips that definitely needed surgery (RMP≥50%) with a sensitivity, specificity and accuracy of 93.4, 97.7 and 97.0, respectively.

Table 6 compares the run-time of both the extended and extended-fast systems for the three-fold cross-validation experiments for all 3300 hips. The results show that the run-time optimised extended-fast system takes on average about one minute, which is less than a fifth of the time of the extended model.

## 4 Discussion

We have developed and evaluated an automatic software system to analyse pelvic radiographs of children with CP, aiming to enhance efficiency and equality of hip surveillance in this population. Our results show moderate to excellent agreement between clinicians and our automatic system. Agreement amongst clinicians was generally within the range of the agreement between clinicians and our automatic system. Agreement for the 50 shared images was similar to the overall results, indicating consistency and reliability of our analysis across subsets of data and clinical experts.

Excellent agreement was achieved for RMP, which is the key clinical measurement for assessing hip migration in children with CP. In contrast, the agreement for AcI was low both across clinicians and between clinicians and our automatic system, indicating potential challenges in accurately measuring AcI in this population. It is noteworthy that for AcI, agreement between clinicians and our automatic system was better than across clinicians. For NSA and HSA, the extended model with the additional femoral shaft points improved the agreement substantially. The worse agreement for HSA compared to NSA, both for manual and automatic measurements, may be due to its dependency on the femoral head growth plate which can be difficult to assess.

The MAD results show acceptable levels of absolute error for all measurements. For RMP in particular, the MAD of 5.7% was within the range of what would be expected if one was to ask clinical experts to manually measure the same image twice (5.8%)^13^. The results from the LMM show that the system has no statistically significant bias when estimating any of the measurements.

The diagnostic consistency results indicate high consistency between the clinicians and the automatic system for diagnoses based on RMP. The high sensitivities and specificities across various thresholds, especially for the extended system, underscore the potential utility of our system in contributing to accurately identifying hips requiring surgical intervention.

Several prior works have approached the problem of automatically assessing hip dysmorphia from radiographs using deep learning. Pham at al^14^ calculated RMP by using deep learning to locate relevant anatomical landmarks. On a small test set of 55 images, their reported ICC values are comparable to ours while their reported MAD results of 4.5% and 4.9% are slightly better. However, they did not compare their system to clinical measurements but rather to measurements obtained by the same researchers that also generated the ground truth to train the deep learning system. So, their system was assessed for how well it compares to the ground truth rather than to clinically obtained measurements. It should also be noted that results are not directly comparable as different datasets were used. Xu at al^15^ used CNN heatmap regression to predict several indicators relevant to hip dysplasia in children with DDH, including AcI. However, they focussed on DDH-related classification performance and did not report on the accuracy of the measurements. Liu at al^16^ used deep learning to measure AcI and detect hip dislocation in children with DDH. Their MAD values for AcI are superior to ours. However, they were obtained in a different patient population which was much younger than ours (with ages from 1 month to 6 years) and which brings less complications in patient positioning during image acquisition affecting the radiographic measurements. Li at al^17^ used deep learning to locate the landmarks necessary to calculate Sharp’s angle, an indicator of hip dysplasia in children with DDH. Archer at al^18^ used a deep learning model to retrieve anatomical landmarks that were then used to calculate several measurements associated with hip dysplasia in adults.

The significant reduction in run-time achieved by the extended-fast system presents a practical advantage, enabling efficient and timely assessment of hip morphology. However, depending on the implementation of such a system as part of the clinical workflow, the expedited run-time may not be necessary. We consider the extended system the key outcome of this work with the extended-fast system an alternative option for when run-time is of importance.

While the system has been assessed on a large clinical dataset of children with CP, all CP data was obtained from a single hospital. To further confirm generalisability of performance, future work will focus on validating the system on CP data from additional hospitals. Initial pilot results on data from Scotland are promising^19^. Further, clinically collected hip radiographs of children with CP sometimes are of poor quality to the extent that measuring the hip is difficult or impossible, even for clinicians (e.g. due to incorrect patient positioning or image artefacts). A limitation of the presented work is that this has not been taken into account and results across all images have been included, though this does increase the generalisability of the findings and better demonstrates their value in clinical practice. However, in future work, we will extend the system to identify when an image is of poor quality or when the result of the automatic point placement is not good enough, to alert the user to intervene. Even though this is likely to only be the case for a small subset of images, it will further improve the clinical applicability and accuracy of the system.

Overall, the findings of this study validate the effectiveness and reliability of the developed system, and highlight the potential impact that this could have in supporting treatment decisions in children with CP. The proposed system has the potential to save clinician’s time and to enable more comprehensive, consistent, and reliable monitoring of hip migration, which could improve outcomes for children with CP. In addition, such a system could facilitate the implementation and increase the uptake of hip surveillance programmes for children with CP.

To facilitate further research, the developed automatic system to locate the point positions will be made available via the BoneFinder® website (https://bone-finder.com).

## Figures and Tables

**Table 1:** Mean absolute deviation (MAD) with standard deviation (SD) for comparing the mean manual and automatic measurements (non-extended and extended systems) across Sets 1-4 (1650 images, left and right hips) for RMP, AcI, NSA and across Set 4 (450 images, left and right hips) for HSA.

	Non-extended	Extended	Extended-fast
**RMP**	5.7% (SD: 8.5%)	5.7% (SD: 8.5%)	5.9% (SD: 9.3%)
**AcI**	4.4° (SD: 5.4°)	4.3° (SD: 5.4°)	4.3° (SD: 5.4°)
**NSA**	6.6° (SD: 6.4°)	5.0° (SD: 5.2°)	5.2° (SD: 5.7°)
**HSA**	7.2° (SD: 6.8°)	5.7° (SD: 6.1°)	6.1° (SD: 6.3°)

**Table 2:** ICC2 scores and 95% confidence bounds quantifying the agreement within each group of clinicians (inter-rater) and between the mean measurement and the automatic measurements (non-extended and extended systems) for the universal shared dataset and Sets 1-4.

	Inter-rater	Non-extended	Extended	Extended-fast
***Universal Set (50 images, 20 clinicians)***				
**AcI**	0.56 (0.51-0.61)	0.69 (0.63-0.74)	0.68 (0.62-0.73)	0.70 (0.64-0.75)
**RMP**	0.89 (0.87-0.90)	0.93 (0.91-0.94)	0.95 (0.94-0.96)	0.91 (0.89-0.92)
**NSA**	0.85 (0.82-0.87)	0.74 (0.61-0.82)	0.87 (0.84-0.89)	0.82 (0.78-0.85)
***Set 1 (450 images, 5 clinicians)***				
**AcI**	0.70 (0.67-0.73)	0.68 (0.65-0.72)	0.68 (0.64-0.72)	0.66 (0.62-0.69)
**RMP**	0.93 (0.92-0.94)	0.93 (0.92-0.94)	0.93 (0.92-0.94)	0.93 (0.91-0.94)
**NSA**	0.90 (0.88-0.91)	0.78 (0.75-0.81)	0.87 (0.85-0.89)	0.85 (0.83-0.87)
***Set 2 (450 images, 5 clinicians)***				
**AcI**	0.53 (0.47-0.59)	0.59 (0.49-0.66)	0.61 (0.51-0.68)	0.62 (0.53-0.69)
**RMP**	0.91 (0.90-0.92)	0.90 (0.88-0.91)	0.91 (0.90-0.92)	0.89 (0.87-0.90)
**NSA**	0.89 (0.87-0.90)	0.74 (0.64-0.80)	0.85 (0.83-0.87)	0.81 (0.79-0.83)
***Set 3 (450 images, 5 clinicians)***				
**AcI**	0.56 (0.45-0.65)	0.69 (0.64-0.73)	0.67 (0.62-0.72)	0.69 (0.64-0.73)
**RMP**	0.89 (0.87-0.91)	0.89 (0.88-0.90)	0.90 (0.89-0.91)	0.88 (0.86-0.89)
**NSA**	0.86 (0.84-0.88)	0.67 (0.55-0.75)	0.83 (0.81-0.85)	0.82 (0.80-0.84)
***Set 4 (450 images, 5 clinicians)***				
**AcI**	0.55 (0.49-0.60)	0.68 (0.64-0.71)	0.67 (0.64-0.71)	0.67 (0.63-0.70)
**RMP**	0.91 (0.88-0.93)	0.91 (0.90-0.92)	0.89 (0.87-0.90)	0.87 (0.86-0.89)
**NSA**	0.88 (0.86-0.90)	0.76 (0.70-0.81)	0.84 (0.82-0.86)	0.82 (0.80-0.84)
**HSA**	0.76 (0.73-0.78)	0.64 (0.53-0.72)	0.73 (0.70-0.76)	0.71 (0.67-0.74)

**Table 3:** ICC1 scores and 95% confidence bounds quantifying the agreement within all of the clinicians (inter-rater) and between the mean measurement and the automatic measurements (non-extended and extended systems) across all four sets (1650 images, left and right hips).

	Inter-rater	Non-extended	Extended	Extended-fast
**AcI**	0.58 (0.57-0.60)	0.66 (0.64-0.67)	0.66 (0.64-0.67)	0.66 (0.64-0.67)
**RMP**	0.91 (0.91-0.91)	0.91 (0.90-0.91)	0.91 (0.90-0.91)	0.89 (0.89-0.90)
**NSA**	0.88 (0.88-0.89)	0.74 (0.72-0.75)	0.85 (0.84-0.86)	0.83 (0.82-0.84)

**Table 4:** LMM bias estimates for comparing the manual measurements and the automatic measurements (non-extended and extended systems) across Sets 1-4 (1650 images, left and right hips) for RMP, AcI, NSA and across Set 4 (450 images, left and right hips) for HSA.

	Non-extended		Extended		Extended-fast	
	Bias	p	Bias	p	Bias	p
**AcI**	-1.37	0.04	-1.33	0.05	-1.33	0.05
**RMP**	0.57	0.41	0.50	0.47	0.57	0.41
**NSA**	-2.9	0.00	0.20	0.43	0.31	0.24
**HSA**	-3.6	0.00	-1.19	0.24	-1.11	0.27

**Table 5:** Diagnostic consistency results comparing the mean manual measurements to the automatic system for diagnoses based on RMP above 30%, 40% and 50% for 3300 CP hips. The results show the percentage of true positives (TP), true negatives (TN), false positives (FP), and false negatives (FN), as well as sensitivity (Sens.), specificity (Spec.) and accuracy (Acc.). Equivalent results reported by Pham et al. [15] are also shown, though these were based on a different dataset and therefore are not directly comparable.

Threshold (%)	TP (%)	TN (%)	FP (%)	FN (%)	Sens. (%)	Spec. (%)	Acc. (%)
***Non-extended***							
**30**	34.7	53.5	8.3	3.0	92.0	86.6	88.7
**40**	22.3	71.1	4.2	2.2	90.9	94.4	93.5
**50**	15.6	81.0	1.9	1.3	92.1	97.7	96.8
***Extended***							
**30**	34.6	53.6	8.1	3.1	91.8	86.8	88.7
**40**	22.6	71.6	3.8	2.0	92.0	95.0	94.2
**50**	15.8	80.9	1.9	1.1	93.4	97.7	97.0
***Extended-fast***							
**30**	34.4	53.5	8.3	3.3	91.4	86.6	88.4
**40**	22.3	70.9	4.4	2.2	91.0	94.1	93.4
**50**	15.3	80.6	2.3	1.6	90.5	97.3	96.1
***[15]***							
**30**	39.1	51.8	3.6	5.5	87.8	93.4	90.9
**40**	10.9	78.2	4.5	6.4	63.2	94.5	89.1

**Table 6:** Average runtime per hip in seconds for both the extended and extended-fast systems for the three-fold cross-validation experiments over all 3300 hips from 1650 images. The timings are when running on a single thread for comparison purposes. Significant speed-up can be obtained by taking advantage of parallel processing.

	Extended (s)	Extended-fast (s)
**Fold1**	333	60
**Fold2**	335	67
**Fold3**	340	55
**Avg.**	336	61

## Take home messages

- The developed system to automatically assess hip migration from pelvic radiographs was evaluated using a large clinical dataset with a wide range of hip displacement and deformity.
- For Reimer’s migration percentage, the most important measurement for hip surveillance in children with cerebral palsy, the agreement between the automatic system and clinical experts was excellent.
- The performance of the automatic system is sufficient for it to be used in clinical practice to assess hip migration in children with cerebral palsy.

## References

[1] Pruszczynski B, Sees J, Miller F (2016). Risk factors for hip displacement in children with cerebral palsy: systematic review. Journal of Pediatric Orthopaedics.

[2] Terjesen T, Horn J (2022). Risk factors for hip displacement in cerebral palsy: A population-based study of 121 nonambulatory children. Journal of Children’s Orthopaedics.

[3] Elkamil AI, Andersen GL, Hägglund G, Lamvik T, Skranes J, Vik T (2011). Prevalence of hip dislocation among children with cerebral palsy in regions with and without a surveillance programme: a cross sectional study in Sweden and Norway. BMC musculoskeletal disorders.

[4] Jeglinsky I, Alriksson-Schmidt AI, Hägglund G, Ahonen M (2022). Prevalence and treatment of hip displacement in children with cerebral palsy in Finland. Journal of Children’s Orthopaedics.

[5] Wordie SJ, Robb JE, Hägglund G, Bugler KE, Gaston MS (2020). Hip displacement and dislocation in a total population of children with cerebral palsy in Scotland: status after five years’ hip surveillance. The Bone & Joint Journal.

[6] Thompson P, Perry DC, Cootes TF, Lindner C, Medical Annotation Collaborative (2012). Automation of Clinical Measurements on Radiographs of Children’s Hips.

[7] Lindner C, Bromiley PA, Ionita MC, Cootes TF (2015). Robust and Accurate Shape Model Matching Using Random Forest Regression-Voting. IEEE Trans Pattern Anal Mach Intell.

[8] Lindner C, Thiagarajah S, Wilkinson JM, Wallis GA, Cootes TF, arcOGEN Consortium (2013). Fully Automatic Segmentation of the Proximal Femur Using Random Forest Regression Voting. IEEE Trans Med Imaging.

[9] Koo TK, Li MY (2016). A guideline of selecting and reporting intraclass correlation coefficients for reliability research. Journal of chiropractic medicine.

[10] Cornell MS, Hatrick NC, Boyd R, Baird G, Spencer JD (1997). The Hip in Children With Cerebral Palsy: Predicting the Outcome of Soft Tissue Surgery. Clinical Orthopaedics and Related Research.

[11] Wordie SJ, Bugler KE, Bessell PR, Robb JE, Gaston MS (2021). Hip displacement in children with cerebral palsy. Bone Joint J.

[12] Faccioli S, Sassi S, Corradini E, Toni F, Kaleci S, Lombardi F (2022). A retrospective cohort study about hip luxation in non-ambulatory cerebral palsy patients: The point of no return. J Child Orthop.

[13] Parrott J, Boyd R, Dobson F, Lancaster A, Love S, Oates J (2002). Hip Displacement in Spastic Cerebral Palsy: Repeatability of Radiologic Measurement. Journal of Pediatric Orthopaedics.

[14] Pham T-T, Le M-B, Le LH, Andersen J, Lou E (2021). Assessment of hip displacement in children with cerebral palsy using machine learning approach. Medical & Biological Engineering & Computing.

[15] Xu W, Shu L, Gong P, Huang C, Xu J, Zhao J (2021). A deep-learning aided diagnostic system in assessing developmental dysplasia of the hip on pediatric pelvic radiographs. Frontiers in Pediatrics.

[16] Liu C, Xie H, Zhang S, Mao Z, Sun J, Zhang Y (2020). Misshapen pelvis landmark detection with local-global feature learning for diagnosing developmental dysplasia of the hip. IEEE Trans Med Imaging.

[17] Li Q, Zhong L, Huang H, Liu H, Qin Y, Wang Y (2019). Auxiliary diagnosis of developmental dysplasia of the hip by automated detection of sharp’s angle on standardized anteroposterior pelvic radiographs. Medicine.

[18] Archer H, Reine S, Alshaikhsalama A, Wells J, Kohli A, Vazquez L (2022). Artificial intelligence generated hip radiological measurements are fast and adequate for reliable assessment of hip dysplasia: an external validation study. Bone & Joint Open.

[19] Hughes K, Lang J, Lindner C, Cootes T, Perry D, Gaston M (2024). Comparing Fully Automated Measurement of Reimer’s Migration Percentage to Manual and Partially Automated methods from Pelvic Radiographs of Children with Cerebral Palsy.

